# 

**Published:** 1846-06

**Authors:** 


QUARTERLY.

PUBLISHED UNDER THE AUSPICES OF THE

**American Society of Dental Surgeons.**

EDITORS:

CHAPIN A. HARRIS M. D., D. D. S.

AMOS WESTCOTT, M. D., D. D. S.

EDWARD MAYNARD, M. D., D. D. S.

BALTIMORE:

ARMSTRONG & BERRY.

JOHN W. WOODS, PRINTER.

$5 00 per annum, payable in advance.

